# Does Mediterranean Adequacy Index Correlate with Cardiovascular Events in Patients with Advanced Chronic Kidney Disease? An Exploratory Study

**DOI:** 10.3390/nu14091687

**Published:** 2022-04-19

**Authors:** Andreana De Mauri, Deborah Carrera, Matteo Vidali, Marco Bagnati, Roberta Rolla, Sergio Riso, Doriana Chiarinotti, Massimo Torreggiani

**Affiliations:** 1 Nephrology and Dialysis Unit, Maggiore della Carità University Hospital, 28100 Novara, Italy; doriana.chiarinotti@maggioreosp.novara.it; 2 Nutritional Science and Dietetic, Maggiore della Carità University Hospital, 28100 Novara, Italy; deborah.carrera@libero.it (D.C.); sergio.riso@maggioreosp.novara.it (S.R.); 3 Clinical Chemistry Unit, Fondazione IRCCS Ca’ Granda Maggiore Policlinico Hospital, 20122 Milano, Italy; matteo.vidali@gmail.com; 4 Clinical Chemistry Laboratory, Maggiore della Carità University Hospital, 28100 Novara, Italy; marco.bagnati@maggioreosp.novara.it (M.B.); robert.rolla@med.uniupo.it (R.R.); 5 Department of Health Sciences, Amedeo Avogadro University of Eastern Piedmont, 28100 Novara, Italy; 6 Néphrologie et Dialyse, Centre Hospitalier Le Mans, 72037 Le Mans, France; maxtorreggiani@hotmail.com

**Keywords:** Mediterranean Adequacy Index, Mediterranean diet, chronic kidney disease, adherence, p-cresol-sulphate, indoxyl-sulphate, Lipoprotein-associated phospholipase A_2_

## Abstract

The Mediterranean Diet (MD) is a healthy dietary pattern, demonstrated to reduce the risk of cancer, diabetes, cardiovascular and neurodegenerative diseases, and early death. The Mediterranean Adequacy Index (MAI) is used to measure adherence to the MD in perspective studies in the general population and correlates with cardiovascular events. The aim of this study was to calculate the MAI among patients with advanced chronic kidney disease (CKD) and correlate it with traditional uremic, microbiota-derived, and proatherogenic toxins as well as nutritional status, quality of life, and cardiovascular events. A total of 60 adult patients with advanced CKD were enrolled and their MAI was calculated. According to the median value, patients were divided into lower (l-MAI, <1.80) and higher (h-MAI, ≥1.80) MAI groups. Biochemical parameters, microbiota-derived and proatherogenic toxins (p-Cresyl sulphate, Indoxyl-sulphate, and Lipoprotein-associated phospholipase A_2_), nutritional status, quality of life, and cardiovascular events that occurred in the previous three years were recorded. The mean value of the MAI was 2.78 ± 2.86. The MAI was significantly higher in foreigners (median (IQR) 6.38 (8.98) vs. 1.74 (1.67), *p* < 0.001) and diabetic patients. The l-MAI and h-MAI groups had similar routinary blood, p-Cresyl-sulphate, Indoxyl-sulphate, and Lp-PLA_2_ as well as nutritional status and quality of life parameters. The MAI was not associated with previous cardiovascular events and did not correlate with cardiovascular events in CKD patients. New and nephro-tailored indexes are warranted to evaluate nutritional therapy in CKD patients.

## 1. Introduction

An association between dietary habits and disease has been described for cardiovascular diseases, diabetes mellitus, healthy aging, breast cancer, colon cancer, cognitive functions, pregnancy and lactation, SARS-CoV-2 infection, and mortality for any cause [1,2,3,4,5,6,7,8,9,10]. While several dietary patterns are now recognized to have a role in health (DASH, Nordic, or vegetarian diet, for instance), the most studied one is the Mediterranean Diet (MD). The scientific interest in the MD began in the early 1960s, with publications arising from the Seven Countries Study [11,12,13]. In 2013, the MD was inscribed on the Representative List of the Intangible Cultural Heritage of Humanity because it involves a set of skills, knowledge, rituals, symbols, and traditions concerning crops, harvesting, fishing, animal husbandry, conservation, processing, cooking, and, particularly, the sharing and consumption of food. Moreover, the MD is an affirmation and renewal of the identity of a family, a group, or a community. The MD emphasizes the values of hospitality, neighborliness, intercultural dialogue, and creativity [14].

Recent reviews confirmed that the MD could lead to a decreased risk of cardiovascular diseases and diabetes, overall cancer incidence, neurodegenerative and dermatological diseases, and early death; the MD could also improve overall health status and reduce total costs of living and costs of national healthcare [15,16,17,18].

As illustrated in the MD pyramid, the traditional MD pattern is characterized by the high consumption of fresh fruits, vegetables, cereals, mainly whole grains, nuts, potatoes, beans, seeds, and extra virgin olive oil as an important source of monounsaturated fats; low-moderate consumption of dairy products, poultry, fish, and wine, mainly red; seldom consumption of red meat, sugar, and sweets; regular physical activity; adequate rest and conviviality [19]. 

Based on these considerations, the nephrological community proposed a role for the MD in chronic kidney disease (CKD), especially in the early stages of the disease, yet the literature is not exhaustive [20,21]. Arguments in favor of prescribing the MD to patients affected by CKD are: a provision of a reduced content of proteins (0.8 g/kg/day), mainly from vegetables, fish, and white meat; a lower load of sodium, potassium, and acids; less atherogenic lipid intake (50% lipid-derived energy from monounsaturated fatty acids, 25% from polyunsaturated and 25% from saturated fatty acids); antioxidant substances from red wine (e.g., resveratrol), and olive oil (vitamin C, E, glutathione, folate); local and eco-friendly production with limited use of processed foods rich in sodium, phosphorus, potassium, and preservatives; and, finally, a high fiber intake. Indeed, the MD provides 30 to 50 g/day of fiber with a 1:1 ratio between soluble and insoluble ones. Fiber has important health-promoting properties, in fact, it lowers sugar and lipid absorption, contributes to body weight control and reduction of the inflammatory status, and modulates microbiota [20]. The intestinal microbiota of CKD patients is characterized by the shift from saccharolytic species to proteolytic ones and generates several toxins [22,23]. Hepatic sulfation of tyrosine and phenylalanine phenolic metabolites leads to the generation p-Cresyl Sulphate (PCS), while the hepatic sulfation of tryptophan generates Indoxyl-sulphate (IS), the two most studied microbiota-derived toxins [24,25]. PCS and IS correlate with the progression of renal failure and with cardiovascular morbidity and mortality in CKD [26,27]. Finally, Lipoprotein-associated phospholipase A_2_ (Lp-PLA_2_) plays a pivotal role in the accelerated atherosclerosis characteristic of uremia [28]. When Lp-PLA_2_ is produced by activated monocytes and macrophages, it enters the vessel wall and induces the chemotaxis of leucocytes into the sub-intimal space. This, in turn, contributes to the instability of the atherosclerotic plaque [28]. As matter of fact, Lp-PLA_2_ predicts acute cardiovascular events [29,30].

As the MD is recognized as a health provider in the general population, tools to measure adherence to the MD were elaborated for epidemiological and clinical research [31]. Among several indexes, the Mediterranean Adequacy Index (MAI) is the most used for observational and prospective studies. It was developed in 1999, to define the characteristics and healthiness of the MD followed in 1960 in Nicotera, one of the pilot villages of the Seven Countries Study [32]. The MAI is easy to calculate by dividing the sum of the percentage of energy from foods typical of the MD by the sum of the percentage of energy from foods not typical of the MD [33]. While the literature largely agrees that the MAI correlates with long-term morbidity and mortality in the general population, no data are available about the eventual role of the MAI in patients with chronic kidney disease [34,35,36]. Likewise, the effect of dietary interventions in advanced CKD on CV outcomes has never been demonstrated.

The aim of this study was, first, to evaluate the MAI among patients with advanced renal failure and, second, to correlate the MAI with traditional uremic, microbiota-derived, and proatherogenic toxins with nutritional status, quality of life, and cardiovascular events in CKD patients. 

## 2. Patients and Methods

### 2.1. Participants

Eligible patients were subjects, older than 18 years, who presented an eGFR lower than 25 mL/min/1.73 m^2^, not on dialysis, not transplanted, and consulted in the outpatient’s ambulatory division of the Nephrology and Dialysis unit at Maggiore della Carità Hospital in Novara. Exclusion criteria were denial or impossibility to sign the informed consent, presence of dementia, history of limb amputation, refusal of the dietary evaluation, state of dialysis, and previous kidney transplant.

Baseline demographic and clinical data, as well as comorbidities, were obtained by reviewing medical notes, clinical summaries, and patient interviews. The following comorbidities were considered: diabetes mellitus, defined as the current or past use of oral hypoglycemic agents or insulin; coronary artery disease (CAD), defined by a history of myocardial infarction, angina, and/or instrumental evidence of ischemic heart disease (electrocardiogram, echocardiogram, stress test, angiography/angioplasty, coronary artery bypass grafting); peripheral artery disease, defined by previous lower limb angioplasty or surgical revascularization or by the presence of clinical signs/symptoms such as intermittent claudication or lower limb extremity ischemic lesions; cerebral disease, defined by the history of a transient or permanent ischemic accident; and hypertension, defined as a blood pressure greater than 140/90 mmHg or the need to use antihypertensive medications to reach an optimal blood pressure control. The presence of an acute cardiac, peripheral, and cerebral vascular event during the 36 months before the enrolment was recorded. 

### 2.2. Assessment of Energy Intake and Diet 

At the first dietary evaluation, a trained dietitian investigated dietary habits by means of a 24 h recall dietary journal. 

By using the Italian food composition tables, the total energy intake (kilocalories per day) was calculated for every patient [37]. 

The MAI was obtained by dividing the sum of the total energy intake percentage of 10 food groups of the reference Mediterranean diet (bread, cereals, legumes, potatoes, vegetables, fresh fruit, nuts, fish, wine, vegetable oils) by the sum the total energy intake percentage of eight food groups less typical of the Mediterranean diet (milk and dairy products, meat, eggs, animal fats and margarine, sweet beverages, cakes and cookies, sugar), as follows:

$$MAI=\frac{\%of\;energy\;bread+cereals+legumes\;dry\;and\;fresh+potatoes+vegetables+fresh\;fruit+nuts+fish+wine+vegetable\;oils}{\%of\;energy\;milk+dairy\;products+meat+eggs+animal\;fats\;and\;margarine+sweet\;beverages+cakes,\;pies\;and\;cookies+sugar}$$

The following laboratory tests were performed on an ADVIA^®^ 1800 Clinical Chemistry Analyzer (Siemens Healthcare Diagnostics, Munich, Germany): urea, creatinine, estimated glomerular filtration rate (eGFR) according to the CKD-EPI equation [38], sodium, potassium, uric acid, calcium, phosphate, parathyroid hormone (PTH), bicarbonate, albumin, hemoglobin, and the total urine sodium excretion. Total urine nitrogen (TUN) excretion was calculated according to the Maroni-Mitch formula [39]:

TUN = urine urea (g/day) + 0.031 × body weight.

Protein Catabolic Rate (PCR) was calculated according to the formula:

PCR = 6.25 × TUN (g/day).

High-performance liquid chromatography coupled with tandem mass spectrometry (B.S.N. Srl, Castelleone, Italy) was used to measure total and free serum p-Cresyl Sulphate (t- and f-PCS) and total and free serum Indoxyl Sulphate (t- and f-IS); serum Lp-PLA_2_ activity was measured with the new PLAC^®^ test (Diazyme Laboratories, Inc., 12889 Gregg Court, Poway, CA 92026, USA). 

Nutritional status was assessed by physical examination, measuring body weight, height, BMI (kg/m^2^), and dominant Hand Grip strength (kg) using Hydraulic Hand Dynamometer Owner’s Manual (Sammons Preston, Bolingbrook, IL, USA), according to the reference values [40,41]. Fat-free body mass (kg), fat mass (kg), and phase angle were determined by means of a bioelectrical impedance analysis (BIA) with an Akern model 101 (Akern Srl, Pisa, Italy).

Quality of life was assessed by means of the Short Form-36 (SF-36), a questionnaire validated and widely used in nephropathic subjects [42,43].

### 2.3. Statistical Analysis

Statistical analyses were performed with SPSS statistical software v.17.0 (SPSS Inc., Chicago, IL, USA). Normal distribution was preliminarily assessed by the Shapiro–Wilk test. Quantitative variables were expressed as the median and interquartile range (IQR), while qualitative variables as absolute and relative frequencies. Patients were divided into two groups: subjects with a MAI lower (l-MAI) and higher (h-MAI) than the median value. Biochemical parameters, nutritional status, and quality of life assessment were compared between the two groups. Differences between the groups were estimated with a nonparametric Mann–Whitney U-test for continuous variables. A correlation was assessed by non-parametric Spearman’s test. Predictors of cardiovascular events: age, MAI (used as continuous variables), and sex, were estimated by multivariate logistic regression. A *p* < 0.05 was considered statistically significant.

## 3. Results

Of the 87 eligible patients, 27 were excluded (8 refused the dietary evaluation, 7 refused to sign the informed consent, and 12 did not satisfy the inclusion criteria) and 60 were enrolled. The median age was 68 (17) years, 70% were males, 90% presented arterial hypertension, 28% had type II diabetes mellitus, and 20% had coronary artery disease (Table 1).

The mean ± standard deviation and median value of the MAI were 2.78 ± 2.86 and 1.80, respectively. 

The MAI was higher among foreigners (No. 6, 10%) than Italian patients (6.38 (8.98) vs. 1.74 (1.67), *p* < 0.001) and among diabetic (No. 17, 28.3%) than nondiabetic patients (2.76 (2.15) vs. 1.73 (1.64), *p* = 0.036).

The MAI did not show any difference between male and female patients (1.95 (2.16) vs. 1.75 (2.47), *p* = 0.705), patients younger or older than 60 years (1.66 (2.53) vs. 1.81 (2.19), *p* = 0.999), and patients with less and more than two comorbidities (1.77 (1.69) vs. 1.83 (2.37), *p* = 0.402).

### 3.1. Comparison in Nutritional Status and Quality of Life

Enrolled patients had, on average, good nutritional status, and no significant variations were observed in both groups, except for pain, which was higher in the h-MAI group (61 vs. 74, *p* = 0.03) (Table 2).

### 3.2. Comparison in Biochemical Parameters 

As illustrated in Table 3, no differences were found in routine blood and urine tests, in PCS, IS, and Lp-PLA_2_, between patients with a MAI lower or higher than the median value of 1.80.

The declared daily protein intake and daily energy intake were lower in subjects with a higher MAI (0.86 vs. 0.70 g/kg/day, *p* = 0.02 and 24.6 vs. 21.4 Kcal/kg/day, *p* = 0.03, respectively). However, the normalized protein catabolic rate, calculated on the urine nitrogen output, did not differ between the two groups (0.90 vs. 0.94, *p* = 0.59). 

### 3.3. Association with Cardiovascular Events

No correlations were found between the MAI and biochemical parameters, nor with PCS or IS, neither with Lp-PLA_2_, nor with the comorbidities at enrollment (Table 4). Serum calcium showed a weak positive correlation with MAI (Table 4).

The multivariate logistic regression analysis showed that neither the MAI (OR 0.703, 95%CI 0.424–1.167, *p* = 0.173), nor age (*p* = 0.077) or sex (*p* = 0.699) were independently associated to CV events in CKD patients (Table 5).

## 4. Discussion

This study demonstrates that the MAI is low in Italian patients with advanced renal failure compared to what is considered adequate in the general population to confer protection from cardiovascular risk [35,44]. Moreover, our study shows that the MAI does not correlate with actual levels of serum uremic, microbiota-derived, and proatherogenic toxins, nor with nutritional status or quality of life, and that the MAI is not associated with cardiovascular events in this particular population of CKD patients. 

The mean and median MAI values of 2.74 and 1.80, respectively, are concordant with previous reports from Italy. After the first value of 7.5 recorded in Nicotera in 1960, subsequent MAIs were much lower in the same years in other villages (2.9 in Crevalcore in 1965 and 5.6 in Montegiorgio in 1965) and in recent years in the same villages (2.2 in Crevalcore in 1991 and 2.4 in Montegiorgio in 1991) [33]. In a larger and more recent survey, the worldwide MAI from 2000 to 2003 was 2.03, with the lowest value recorded in northern Europe (0.85) and the highest one in non-European Mediterranean countries (2.49) [45]. In this latter study, the Italian MAI significantly decreased from 3.30 in 1961–1965 to 1.62 in 2000–2003 [45]. According to the analysis of da Silva and colleagues, the median Italian MAI was 3 for men and 2.4 for women. These data were generated in a study from Molise, a rural region of central Italy and these figures may be different in an urban population of a northern industrial Italian region such as Piedmont where our study was conducted [45]. However, it is important to note that in a study from Italy, the average MAI associated with the absence of fatal coronary heart disease at 20 or 40 years of follow-up were 6.8 and 6.9, respectively; much higher than what we found in our population [35].

Among our patients, we found some differences. First, the MAI was much higher in foreigners than in Italian subjects. Among foreigners, we had two patients from Morocco, one from the Ivory Coast, one from Sri Lanka, one from Pakistan, and one vegetarian from Albania. They were all first-generation immigrants and retained their traditional dietary habits typical of Muslim or Buddhist countries with a poor consumption of pork meat and alcohol or with a regimen mainly based on vegetables. This could explain the higher MAI of foreigners compared to Italians. Second, the MAI was higher among diabetic than non-diabetic patients, maybe because diabetic subjects usually receive dietary counseling and are prone to eat less sugar and sweets and more complex carbohydrates.

Curiously, the MAI was similar between males and females; this could be explained by the fact that in the traditional Italian family, women are cooking for the entire family [46]. Furthermore, whereas the MAI was not different between older and younger people, there was a different contribution to the ratio; while the high consumption of traditional and local products, such as red meat, ham, salami, cheese, and animal fats, is more frequent among older people, the consumption of sweet beverages, sweets, cakes, cookies, and pies is more prevalent among younger-ones [47,48]. 

In the present study, no correlations were found between the MAI and the serum level of blood urea nitrogen, phosphorus, lipids, alkali, neither with PC, IS, Lp-PLA_2_, nor with the comorbidities at enrollment. A positive correlation was only found with serum calcium levels, in line with previous observations that associated the MD with higher levels of calcium [49]. This is not an unexpected finding: chronic kidney disease is a very complex pathology and not only a “comorbidity”, thus the plethora of alterations induced by CKD cannot be modulated only by the diet. In addition, even in the MOLI-SAL project, the MAI was not associated with cholesterol, glucose and hypertensive status, or body mass index in healthy screening [50].

Curiously, patients with a lower MAI declared a daily protein intake similar to the value inferred from the normalized protein catabolic rate, while subjects with a higher MAI declared a lower protein intake. Similarly, patients with a higher MAI had a lower daily energy intake as inferred from the dietary recall, but this data could not be verified in the present study. These findings suggest that patients more adherent to the MD may have an incorrect perception of their dietary habits that should be always confirmed by more objective evaluations. Due to the renowned healthiness of the MD, patients could have underestimated their protein and calory intake.

An important finding of the study is the absence of correlation between the MAI and cardiovascular events that occurred in the 3 years before the enrollment. It could be argued that the dietary pattern changed over three years, but it has been shown that people tend to stick to their eating habits in the absence of any specific interventions [51,52,53,54]. Thus, we can assume that the previous three-year dietary pattern was similar to our baseline evaluation. Considering that a MAI greater than 3, corresponding to the minimum MAI in the Mediterranean area in the early 1960s, was demonstrated to have a protective role, our study highlights the need for effective dietary interventions even in a Mediterranean country such as Italy [11,12,33,35,45,55]. 

The present study has some limitations. First, it lacks longitudinal follow-up to observe cardiovascular events, neurodegenerative disorders, cancer, and death incidence. Second, the low number of enrolled patients may limit the appreciation of some expected results in terms of statistical significance. Third, physical activity and smoking status were not analyzed because of incomplete data and low prevalence. Finally, it is likely that patients with poor dietary habits are less prone to participate in such types of studies, thus generating a potential selection bias.

As accepted in the literature, the MAI, as far as it has been validated, presents some limits. It does not indicate the proportions of every component of the diet, nor the energy and the contribution of every component to the total energy which is very important in CKD patients. Again, the MD is different across Mediterranean countries because of the different local and traditional foods, and, consequently, the adherence to the MD is hard to compare. To cope with this issue, other indexes were elaborated to measure the adherence to the MD: the Mediterranean Diet Score to test adherence in the Greek population with high consumption of green wild vegetables; the Mediterranean Score that better distinguishes the metabolic and lipid profile; the Mediterranean Diet Quality index to evaluate the content of carotene, vitamin E, and other anti-inflammatory agents; and the Kidmed to evaluate dietary habits in children [56,57,58,59]. However, two reviews evidenced a low correlation between all these tools in estimating the real adherence to the MD [31,60]. 

Our cohort was composed of patients who had not been receiving specific dietary counseling before but, nonetheless, the low MAI values in our population were quite a surprising finding as we thought that the MD should be part of their cultural heritage, especially for Italian patients. Since the MAI did not correlate with cardiovascular events in CKD subjects, we postulate that the uremic milieu overcomes the protective effects of the MD. Even if, in the general population, a MAI higher than 3.4 is considered adequate, it may not be enough to observe the benefits of the MD in CKD patients. In this context, we propose that the MAI (or its variations) has limited usefulness to estimate the adherence to the MD in CKD, as it does not result in greater protection from CV events in this population. However, considering the widely studied benefits of the MD in the general population, a novel index, or a new and “nephro-tailored” MAI, is warranted to monitor the adherence to and the benefits of the MD in nephropathic subjects.

To date, low protein diets, characterized by a protein intake of 0.6 to 0.2 g per kilogram of body weight per day, that are vegetable-enriched and sodium and phosphorus-depleted, are a cornerstone of CKD management because they delay the progression to end-stage kidney disease and increase patients’ survival [61,62,63,64]. Therefore, due to the complexity of determining compliance, concordance, and adherence to dietary interventions in advanced kidney disease and the different effects these parameters have on the outcomes, nephrologists need more appropriate indexes not only to monitor the MD but also and above all, for tailoring low protein diets in the context of the multidisciplinary approach typical of the management of CKD [65,66,67].

## 5. Conclusions

To the best of our knowledge, this is the first study showing that the MAI is low and is not associated with cardiovascular events in CKD patients. However, due to the exploratory nature of our study, further prospective trials are warranted to confirm our results or determine the predictive potential of the MAI in CKD patients.

## Figures and Tables

**Table 1:** Characteristics of patients.

	*n*. 60
Age, years	68 (17)
Male/Female *n* (%)	42/18 (70/30%)
Hypertension *n* (%)	54 (90%)
Diabetes *n* (%)	17 (28.3%)
Coronary artery disease *n* (%)	12 (20%)
Cerebral vascular disease *n* (%)	11 (18.3%)
Peripheral vascular disease *n* (%)	1 (1.7%)
Cause of renal failure Hypertension *n* (%) Diabetes *n* (%) Genetics *n* (%) Other *n* (%)	24 (40%) 9 (15%) 11 (18%) 16 (27%)
nPCR, g/kg/day	0.93 (0.41)
Declared protein intake, g/kg/day	0.75 (0.28)
Total energy intake, Kcal/kg/day	22.90 (8.30)

Results are expressed as median and interquartile ranges for quantitative variables and as absolute and relative frequencies for qualitative variables. nPCR: normalized protein catabolic rate.

**Table 2:** Comparison between nutritional status and quality of life indicators in low- and high-MAI patients.

	l-MAI	h-MAI	*p*
BMI, kg/cm^2^	28.0 (6.9)	28.0 (7.8)	0.57
Lean Mass, kg	49.9 (15.7)	57.7 (17.7)	0.71
Fat Mass, kg	22.7 (10.8)	23.4 (5.4)	0.74
Angle Phase	4.9 (1.4)	4.9 (1.8)	0.27
Hand Grip, kg	28.0 (18.0)	36 (14.8)	0.09
Physical functioning, points	75 (35)	80 (35)	0.19
Role limitation due to physical health, points	37 (100)	62 (100)	0.59
Pain, points	61 (66)	74 (52)	**0.03**
General Health, points	41 (34)	56 (27)	0.23
Energy-fatigue, points	45 (39)	57 (27)	0.48
Social functioning, points	62 (47)	75 (50)	0.75
Role limitation due to emotional problems, points	66 (100)	83 (100)	0.45
Emotional well-being, points	66 (39)	72 (27)	0.53

Results are expressed as median and interquartile ranges. BMI: body mass index. In bold, significant values.

**Table 3:** Comparison between routine biochemical parameters, microbiota-derived toxins, and Lp-PLA_2_ in low- and high-MAI patients.

	l-MAI	h-MAI	*p*
MAI	1.14 (0.71)	3.40 (1.46)	
Age, years	69 (18)	68 (16)	0.68
eGFR, mL/min	19.40 (4.95)	17.80 (4.90)	0.57
Proteinuria, g/24 h	0.75 (2.62)	1.50 (1.83)	0.74
Hemoglobin, g/dL	11.4 (2.7)	11.7 (2.1)	0.17
BUN, mg/dL	47 (19)	53 (18)	0.97
Uric acid, mg/dL	5.7 (1.2)	6.4 (3.1)	0.72
Albumin, mg/dL	4.2 (0.5)	4.3 (0.5)	0.12
Calcium, mg/dL	9.2 (1.0)	9.3 (0.8)	0.02
Phosphorus, mg/dL	3.6 (0.7)	3.5 (1.3)	0.44
Total cholesterol, mg/dL	176 (57)	179 (42)	0.83
HDL, mg/dL	47 (19)	42 (20)	0.05
Triglycerides, mg/dL	158 (62)	145 (131)	1.00
LDL, mg/dL	99 (59)	100 (49)	0.92
HCO_3_^−^, mEq/L	23.6 (3.7)	22.5 (5.0)	0.65
C-Reactive Protein, mg/dL	0.26 (0.49)	0.22 (0.61)	0.41
PTH, ng/mL	69.1 (85.8)	64.4 (61.9)	0.81
Urinary Sodium, mEq/day	137 (88)	134 (57)	1.00
nPCR, g/kg/day	0.90 (0.34)	0.94 (0.40)	0.59
Declared protein intake, g/kg/day	0.86 (0.43)	0.70 (0.17)	**0.02**
Total energy intake, Kcal/kg/day	24.60 (7.55)	21.40 (7.77)	**0.03**
Lp-PLA_2_, nmol/mL/min	137 (63)	163 (63)	0.92
t-PCS, mcMol	144 (87)	102 (121)	0.43
f-PCS, mcMol	4.67 (3.47)	4.40 (5.38)	0.98
t-IS, mcMol	32.4 (23.3)	24.9 (19.3)	0.16
f-IS, mcMol	1.22 (0.87)	1.21 (0.79)	0.70

Results are expressed as median and interquartile ranges. nPCR: normalized protein catabolic rate. MAI: Mediterranean Adequacy Index; eGFR: estimated glomerular filtration rate; BUN: blood urea nitrogen; HDL: high density lipoprotein; LDL: low density lipoprotein; HCO_3_^−^: bicarbonates; PTH: parathyroid hormone; nPCR: normalized protein catabolic rate; Lp-PLA_2_: Lipoprotein-associated phospholipase A_2_; t-PCS: total p-Cresyl Sulphate; f-PCS: free p-Cresyl Sulphate; t-IS: total Indoxyl-sulphate; f-IS: free Indoxyl-sulphate. In bold, significant values.

**Table 4:** Correlation table.

		Age	eGFR	PTU	Hemoglobin	Urea	Uric Acid	Albumin	Calcium	Phosphate	Total Cholesterol	HDL	Triglycerides
MAI	Correlation coefficient	−0.060	0.002	0.051	0.136	0.078	0.070	0.204	0.314	−0.065	−0.032	−0.202	−0.041
	*p*	0.647	0.989	0.703	0.300	0.552	0.620	0.117	0.015	0.620	0.811	0.121	0.755
	*n*	60	60	59	60	60	52	60	60	60	60	60	60
		**LDL**	**HCO**_**3**_^**−**^	**CRP**	**PTH**	**UNa**	**nPCR**	**t-PCS**	**f-PCS**	**t-IS**	**f-IS**	**Lp-PLA**_**2**_	**Number of Comorbidities**
MAI	Correlation coefficient	−0.032	0.065	−0.021	0.009	0.017	0.201	−0.143	−0.022	−0.232	−0.087	−0.012	0.125
	*p*	0.817	0.628	0.875	0.946	0.900	0.124	0.294	0.875	0.085	0.524	0.932	0.340
	*n*	55	58	60	60	59	60	56	56	56	56	53	60

MAI: Mediterranean Adequacy Index; eGFR: estimated glomerular filtration rate according to the CKD-EPI formula; PTU: proteinuria; HDL: high density lipoprotein; LDL: low density lipoprotein; HCO_3_^−^: bicarbonates; CRP: C-reactive protein; PTH: parathyroid hormone; Una: urinary sodium excretion; nPCR: normalized protein catabolic rate; t-PCS: total p-Cresyl Sulphate; f-PCS: free p-Cresyl Sulphate; t-IS: total Indoxyl-sulphate; f-IS: free Indoxyl-sulphate; Lp-PLA_2_: Lipoprotein-associated phospholipase A_2_.

**Table 5:** Multivariate logistic regression of predictors of cardiovascular events.

		95% CI		
	Odds-Ratio	Lower	Higher	*p*-Value
Age, years	1.064	0.993	1.140	0.077
Sex (males vs. females)	1.311	0.332	5.171	0.699
MAI	0.703	0.424	1.167	0.173

MAI: Mediterranean Adequacy Index.

## Data Availability

The data presented in this study are available on request from the corresponding author.
